# Extracting Drug-Drug Interaction from the Biomedical Literature Using a Stacked Generalization-Based Approach

**DOI:** 10.1371/journal.pone.0065814

**Published:** 2013-06-13

**Authors:** Linna He, Zhihao Yang, Zhehuan Zhao, Hongfei Lin, Yanpeng Li

**Affiliations:** School of Computer Science and Technology, Dalian University of Technology, Dalian, China; Indiana University, United States of America

## Abstract

Drug-drug interaction (DDI) detection is particularly important for patient safety. However, the amount of biomedical literature regarding drug interactions is increasing rapidly. Therefore, there is a need to develop an effective approach for the automatic extraction of DDI information from the biomedical literature. In this paper, we present a Stacked Generalization-based approach for automatic DDI extraction. The approach combines the feature-based, graph and tree kernels and, therefore, reduces the risk of missing important features. In addition, it introduces some domain knowledge based features (the keyword, semantic type, and DrugBank features) into the feature-based kernel, which contribute to the performance improvement. More specifically, the approach applies Stacked generalization to automatically learn the weights from the training data and assign them to three individual kernels to achieve a much better performance than each individual kernel. The experimental results show that our approach can achieve a better performance of 69.24% in F-score compared with other systems in the DDI Extraction 2011 challenge task.

## Introduction

A drug-drug interaction (DDI) occurs when one drug influences the level or activity of another [1]. A patient may take a variety of drugs at one time. However, one drug may influence the others, and sometimes these influences result in side effects that are dangerous to patients. Therefore, DDI detection is important for patient safety. Doctors should not prescribe combinations of drugs that have side effects when taken together. Furthermore, DDI detection is also important for pharmacists; if pharmacists are aware of the interactions that may occur between drugs, they can list these interactions in the specifications so that the patients will know which drugs cannot be taken together.

As drug-drug interactions are frequently reported in journals of clinical pharmacology and technical reports, the biomedical literature is the most effective source for the detection of DDIs [1]. The development of Information extraction (IE) tools for automatically extracting DDIs from the biomedical literature is important to reduce the time that professionals must spend reviewing the relevant literature. Meanwhile, it is essential for improving and updating the drug knowledge databases [1].

In fact, information extraction from the biomedical literature has been a topic of intense investigation during recent years [2]. For example, many kernel-based methods, such as subsequence kernels [3], tree kernels [4], shortest path kernels [5], and graph kernels [6], have been proposed and successfully used to extract protein-protein interactions (PPIs). However, few approaches have been proposed to solve the problem of extracting DDIs from the biomedical texts. Segura-Bedma et al. applied a linguistic rule-based approach to extract DDIs [7]. Then, they proposed another approach called the shallow linguistic (SL) kernel [8] to extract DDIs [1].

In the DDI Extraction 2011 challenge task [9], more approaches were proposed to extract DDIs from the biomedical literature. Thomas et al. [10] used an approach called majority voting ensembles (WBI-5) that consists of three methods, namely the all-paths graph (APG) kernel [6], the shallow linguistic (SL) kernel and Moara, which is an improved system that participated in the BioNLP’09 Event Extraction Challenge [11]. Among others, the APG kernel obtains the best performance with an F-score of 63.53%. When further combined with SL and Moara, it obtains a performance of 65.74% for the F-score, ranking first in the DDI Extraction 2011 challenge task. Moreover, Chowdhury et al. [12] applied different machine learning techniques that include a feature-based method and a kernel-based method consisting of a mildly extended dependency tree (MEDT) kernel [13], a phrase structure tree (PST) kernel [14], and a SL kernel to extract DDIs. The union of the feature-based and kernel-based methods obtains a performance with an F-score of 63.98% ranking second in the task. Björne et al. proposed the Turku Event Extraction system to extract DDIs, and their result ranks fourth with an F-score of 62.99% [15]. What’s more, Minard et al. used only a feature-based kernel that includes many types of features to extract DDIs [16]. Their approach introduces the feature selection according to the features’ F-measure improved interaction detection. As a result, their performance ranks fifth with an F-score of 59.65%. On the whole, the research of DDI extraction from the biomedical literature is still at an early stage, and its performance has much room to improve (in the DDI Extraction 2011 challenge task, the best performance achieved is 65.74% in F-score [10]).

In this paper, we propose a Stacked generalization-based approach [17] to extract DDIs from the biomedical literature. The approach introduces Stacked generalization to automatically learn the weights from the training data and assigns them to three individual kernels, feature-based, tree and graph kernels, and achieves much better performance than each individual kernel. The performance of our approach is superior to those of [10], [12]. The key reasons are as follows: 1) In addition to the commonly used word features, our feature-based kernel introduces the semantic type feature, the keyword feature and the DrugBank (http://www.drugbank.ca/) features. The introduction of these features allows for the utilization of domain knowledge and improves the performance effectively (nearly 1.2 percentage point in the F-score). 2) The features in individual kernels are complementary, and their combination with Stacked generalization can achieve better performance than each individual kernel.

## Methods

A kernel can be thought of as a similarity function for pairs of objects. Different kernels calculate the similarities with different aspects between two sentences. Combining the similarities can reduce the risk of missing important features and thus produces a new useful similarity measure. Our method combines several distinctive types of kernels to extract DDIs: namely, the feature-based, graph and tree kernels.

### Feature-based Kernel

Before the features used in our feature-based kernel are introduced, some notions of words, n-grams, areas and positions in a sentence with regard to two interacting drugs (which are replaced by “drug1” and “drug2” in our experiments) are given.

1. Vocabularies of words: VW = {words in training data}.
2. Vocabularies of word-level n-grams: VN = {1–3 grams in training data}
3. Assume that words in an DDI instance are indexed by the following sequence:

Indices = (0, …, d1, …, d2, …, END).

Where *d1* is the index of the token “drug1”, *d2* is the index of the token “drug2”, and *END* is the index of the last token of the sentence.

1. General areas: GA = {Left_Area, Inner_Area, Right_Area} = {[0, d1–1], [d1+1, d2–1], [d2+1, END]} – text snippets split by “drug1” and “drug2” in each sentence denoted by“Left_Area drug1 Inner_Area drug2 Right_Area”.
2. Surrounding areas: SA = {D1_Left, D1_Right, D2_Left, D2_Right} = {[d1–4, d1–1], [d1+1, d1+4], [d2–4, d2–1], [d2+1, d2+4]} – texts surrounding “drug1” or “drug2” within a 4-word window.
3. Conjunct positions: CP = { D1_direction ^∧^ D2_direction ^∧^ distance | direction 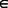
{Left, Right}, distance = discretized (d2-d1) 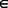
{0,1, 2, 3, 4, 5, (6∼7), (8∼10), (11∼15), (16∼20), (21∼30), (31∼40), (40∼) } – conjunctions of partial elements (two areas adjacent each drug) and the discretized word count between the two drugs.

#### Lexical features

Our lexical features take three unordered sets of words as the feature vectors (an example of the features generated by the feature-based kernel is given in Table 1):

**Table 1:** Features generated in our feature-based kernel for the instance “Plasma concentrations of **drug1** are decreased when administered with **drug2** containing drug0 or drug0.”

Feature	Feature value	
Abon	n = 1	left_area = Plasma, left_area = concentrations, left_area = of; Inner_area = are, Inner_area = decreased,…, Inner_area = with; Right_area = containing,…, Right_area = drug0.
	n = 2	left_area = Plasma concentrations, left_area = concentrations of; Inner_area = are decreased,…, Inner_area = administered with; Right_area = containing drug0,…, Right_area = or drug0.
	n = 3	left_area = Plasma concentrations of; inner_area = are decreased when,…, inner_area = when administered with; Right_area = containing drug0 or; Right_area = drug0 or drug0.
San	n = 1	D1_left = Plasma, D1_left = concentrations, D1_left = of; D1_right = are, D1_right = decreased,…; D2_left = decreased,…; D2_right = containing,…
	n = 2	D1_left = Plasma concentrations, D1_left = concentrations of; D1_right = are decreased, D1_right = decreased when,…; D2_left = decreased when; D2_left = when administered,…; D2_right = containing drug0…
	n = 3	D1_left = Plasma concentrations of; D1_right = are decreased when, D1_right = decreased when administered; D2_left = decreased when administered, D2_left = when administered with;D2_right = containing drug0 or, D2_right = drug0 or drug0
Cpn	n = 1	D1_left^∧^D2_left^∧^distance = Plasma^∧^are^∧^5, D1_left^∧^D2_left^∧^distance = Plasma^∧^ decreased^∧^5, D1_left^∧^D2_left^∧^distance = Plasma^∧^when^∧^5…; D1_right^∧^D2_left^∧^distance = are^∧^are^∧^5…; D1_left^∧^D2_right^∧^distance = Plasma^∧^containing^∧^5…; D1_right^∧^D2_right^∧^distance = are^∧^containing^∧^5…
	n = 2	D1_left^∧^D2_left^∧^distance = Plasma concentrations^∧^are decreased^∧^5,…;D1_right^∧^D2_left^∧^distance = are decreased^∧^are decreased^∧^5,…; D1_left^∧^D2_right^∧^distance = Plasma concentrations^∧^containing drug0^∧^5,…; D1_right^∧^D2_right^∧^ditance = are decreased^∧^containing drug0^∧^5,…
	n = 3	D1_left^∧^D2_left^∧^distance = Plasma concentrations of^∧^are decreased when ^∧^5,…; D1_right^∧^D2_left^∧^distance = are decreased when^∧^are decreased when^∧^5,…; D1_left^∧^D2_right^∧^distance = Plasma concentrations of^∧^containing drug0 or^∧^5,…; D1_right^∧^D2_right^∧^distance = are decreased when^∧^containing drug0 or^∧^5…
Negative word		no = -1, not = −1,…
Keyword		decrease = 1, activate = = −1,…
Semantic type		Semtype1 = “carb,phsu”, semtype2 = “gngm”
NameIsDrug		entity1 = −1 entity2 = −1
DrugBank		indication = 0, pharmacology = 0, description = 0

1. Area-bag-of-n-grams (Abon): features derived from VW×GA and VN×GA. The features of VW×GA, e.g., “*left_area = Plasma*”, ignore word positions in the current area, and the features of VN×GA, e.g., “*left_area = Plasma concentrations*”, simply enrich the bag-of-words representation by bigrams and trigrams.
2. Surrounding area-n-grams (San): features from VN×SA, e.g., “D1_Right = increase the”. They are used to highlight n-grams in the “indicating areas” (i.e., D1_left, D1_right, D2_left and D2_ right) because, intuitively, features surrounding candidate drug pairs are more indicative.
3. Conjunction position n-grams (Cpn): features from VN×CP. The feature set is the conjunctions of a subset of the surrounding position-n-gram features (the features from VN×SP, which gives the information of specific distances from the n-grams in SA to focusing drugs, e.g., “*-1_from_D1 = of*”) and the distance of two drugs, e.g., “*D1_left* ^∧^ *D2_left* ^∧^ *distance = of* ^∧^ *with* ^∧^ *5*”. It can simultaneously capture the lexical information around both drugs. The only problem is that they may suffer from data sparseness.

In addition to the commonly used lexical features, five extra features are introduced into the feature-based kernel.

#### Negative word feature

In most cases, the existence of negative words in a DDI instance denotes that there is no interaction between the two drugs. Therefore, we introduce the negative word feature. The negative words used in our experiment include “no”, “not”, “neither”, “fail”, “fail to”, “fails to”, “failed”, “failed to” and “failure”. If one of the negative words exists in an instance, the feature value will be set to 1; otherwise 0.

#### NameIsDrug feature

In both the training and test sets of the DDI Extraction 2011 challenge task, “drug”, “drugs”, “Drug” and “Drugs” are sometimes labeled as drug names and sometimes not. There are approximately 520 such labeled instances in the training set, as shown in the following example: “The interactions between ***Betaseron*** and other ***drugs*** have not been fully evaluated”. To solve the problem, we introduce the NameIsDrug feature. If only one of the two entities is referred to by the terms “drug” or “drugs” or “Drug” or “Drugs”, we set the feature as “entity1 = 1 entity2 = −1” or “entity1 = −1 entity2 = 1”. Otherwise, the feature is set as “entity1 = 1 entity2 = 1” or “entity1 = −1 entity2 = −1”.

#### Keyword feature

The existence of an interaction keyword (the verb or their variants expressing drug interaction relation, such as “activate,” “activation,” “counteract,” “inhibit,”) between two drug names often implies the existence of the DDI. Thus, it is chosen as a feature in our feature-based kernel. To identify the keywords in texts, we manually created an interaction keyword list of 506 entries, which includes the interaction verbs and their variants.

#### Semantic type feature

The dataset of DDI Extraction 2011 challenge task is made available in two formats: the so-called unified format and the MMTx format. The unified format contains only the tokenized sentences, while the MMTx format contains the tokenized sentences along with the POS tag and the semantic type for each token. An experienced pharmacist reviewed the UMLS Semantic Network as well as the semantic annotation provided by MMTx and recommended the inclusion of the following UMLS semantic types as possible types of interacting drugs [9]: Clinical Drug (clnd), Pharmacological Substance (phsu), Antibiotic (antb), Biologically Active Substance (bacs), Chemical Viewed Structurally (chvs) and Amino Acid, Peptide, or Protein (aapp). Because DDIs may exist between two entities with the specific semantic types, we introduce the semantic type feature, which is extracted from the MMTx format dataset.

#### DrugBank feature

DrugBank database is a rich resource combining chemical and pharmaceutical information for approximately 4,900 pharmacological substances [18]. For each drug, DrugBank contains more than 100 data fields including drug synonyms, brand names, chemical formulas and structures, drug categories, ATC and AHFS codes (i.e., codes of standard drug families), mechanism of action, indication, dosage forms, toxicity, etc.

Based on the idea that the information in the DrugBank fields may help classify the DDI instance, we introduce the DrugBank features. Three DrugBank fields are found through experiments to be helpful for the performance improvement of DDI extraction, namely, Indication, Pharmacology and Description. For the two drugs in one DDI instance, we construct their corresponding N-dimensional feature vectors 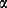 and 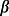 of words using the three DrugBank fields: if a word 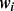
(*i = *1,…,*N*) exists in the DrugBank field of one drug, the corresponding vector element is set to 1; otherwise, 0. Then, the similarity between 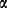 and 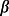 is calculated by the Cosine similarity defined as follows:
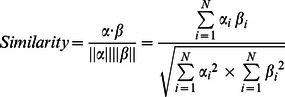
(1)


If the Cosine similarity of one DrugBank field of two drugs is more than or equal to 0.4 (which is determined experimentally), the corresponding feature is set to 1; otherwise 0.

### Graph Kernel

**Figure 1:**
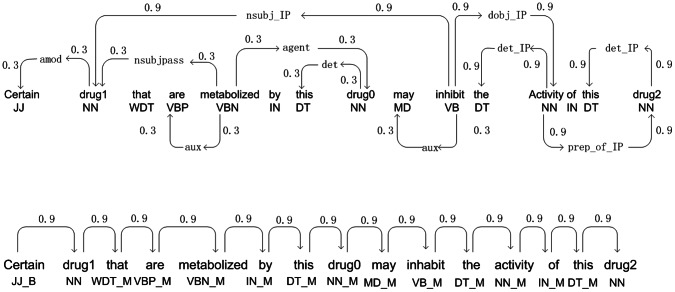
Graph representation generated from an example sentence. The candidate interaction pair is marked as “drug1” and “drug2”, the other drugs are marked as “drug0”. The shortest path between the drugs is shown in bold. In the dependency based subgraph all nodes in a shortest path are specialized using a post-tag (IP). In the linear order subgraph possible tags are (B)efore, (M)iddle, and (A)fter.

A graph kernel calculates the similarity between two input graphs by comparing the relations between common vertices (nodes). The weights of the relations are calculated using all walks (possible paths) between each pair of vertices. Our method follows the all-paths graph kernel proposed by Airola et al. [6]. The kernel represents the target pair using graph matrices based on two sub-graphs, one representing the parse structure sub-graph, and the other representing the linear order sub-graph (see Figure 1). The first sub-graph represents the parse structure of a sentence and includes word or link vertices. A word vertex contains its lemma and POS, while a link vertex contains its link. In addition, both types of vertices contain their positions, which differentiate them from other vertices in Figure 1. The second sub-graph represents the word sequence in the sentence, and each of its word vertices contains its lemma, its relative position to the target pair and its POS.

For the calculation, two types of matrices, specifically edge matrix 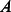 and label matrix 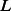, are used. We assume that 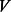 represents the set of vertices in the graph, and 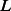 represents the set of possible labels that vertices can have. We represent the graph with an adjacent matrix 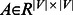 whose rows and columns are indexed by the vertices, and 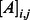 contains the weight of the edge connecting 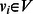 and 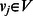 if such an edge exists, and it is 0 otherwise. The weight is a predefined constant whereby the edges on the shortest paths are assigned a weight of 0.9 and other edges receive a weight of 0.3. Additionally, we represent the labels as a label allocation matrix 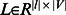 so that 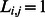 if the *j-th* vertex has the *i-th* label, and 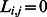 otherwise. Using the Neumann Series, a graph matrix G is calculated as:
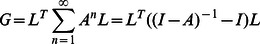
(2)


This matrix sums up the weights of all the walks between any pair of vertices so, as a result, each entry represents the strength of the relation between a pair of vertices. Using two input graph matrices *G*′ and *G*″, the graph kernel *K*(*G*′, *G*″) is the sum of the products of the common relations’ weights, given by Equation (3).
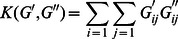
(3)


### Tree Kernel

As a specialized convolution kernel, the tree kernel used in our work aims to capture structured information in terms of substructures. 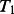 and 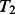 represent two parse trees. The convolution tree kernel 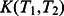 counts the number of common sub-trees as the syntactic structure similarity between two parse trees *T*_1_ and *T*_2_ [19]:
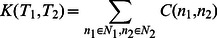
(4)where *N_i_* is the set of nodes in tree *T_i,_* and we note that 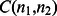 can be computed in polynomial time due to the following recursive definition:

1. If the context-free productions-Context-Free Grammar rules-at 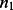 and 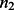 are different, 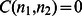;
2. If both 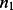 and 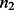 are POS tags, 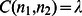; otherwise go to (3).
3. Compute 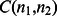 recursively as:

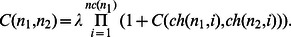
where 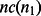 is the number of children of n_1_ in the tree. 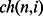 is the *i-th* child of node n and 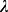 is the decay factor.

#### Parse tree kernel

The parse tree encapsulates the relation instance between two focused entities. Thus, we should know which portion of a parse tree is important in the tree kernel calculation.

A standard tree kernel is often defined on the minimum complete tree (MCT) that contains both candidate entities in a parse tree. The MCT is the complete sub-tree rooted by the node of the nearest common ancestor of the two entities under consideration. However, the MCT may introduce some random noise features. Thus, we introduced the SPT, which is the smallest common sub-tree including the two focused entities.

In some cases, the information contained in the SPT is not sufficient to determine the relationship between two candidate entities. By analyzing the experimental data, we found that in these cases the SPTs usually have fewer than four leaf nodes and, therefore, include little information except the two entity names. Therefore, we employ a simple heuristic rule to expand the SPT span: we adopt SPT as our tree span. If the number of leaf nodes in an SPT is smaller than four, the SPT is expanded to a higher level, i.e., the parent node of the root node of the original SPT is used as the new root node.

#### Dependency path tree kernel

Information of dependency path tree is another type of tree structure information that is provided by the parser dependency analysis output in our tree kernel. For dependency based parse representations, a dependency path is encoded as a flat tree as depicted as follows: (DEPENDENCY (NSUBJ (interacts DRUG1)) (PREP (interacts with)) (POBJ (with DRUG 2))) corresponding to the sentence “DRUG1 interacts with DRUG2”. Because a tree kernel measures the similarity of the trees by counting the common subtrees, it is expected that the system finds effective subsequences of dependency paths. The dependency path tree is similar to SPT, which also needs extension in some cases. By analyzing the experimental data, we determined that if the length of the dependency path between two focus drugs is shorter than three, it is extended. Meanwhile, if two edges are present to the left of the first drug in the whole dependency parse path, they will be included in the dependency path. Otherwise, the two edges to the right of the second drug will be included in the dependency path. The optimal extension threshold 3 is determined through 10-fold cross-validation on the training set. The original training set is randomly partitioned into 10 equal size subsamples. Of the 10 subsamples, a single subsample is retained as the validation data for testing the model, and the remaining 9 subsamples are used as training data. The cross-validation process is then repeated 10 times, with each of the 10 subsamples used exactly once as the validation data. The 10 results from the folds then are averaged to produce a single estimation.

### Combination of Kernels using Stacked Generalization

Each kernel has its own pros and cons. The parse tree kernel does not output certain shallow relations, and conversely, the dependency path kernel ignores some deep information. The feature-based kernel cannot capture the sentence structure. The graph kernel can treat the parser’s output and word features at the same time, but it may miss some distant words and similarities of paths among more than three elements [20].

Each kernel calculates the similarity between two sentences from different aspects. Thus combining the similarities can reduce the risk of missing important features. To realize the combination of different types of kernels based on different parse structures, the normalized output of several kernels K_m_ can be defined as:
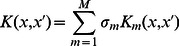
(5)

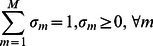
(6)where M represents the number of kernel types, and 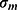 is the weight of each *K_m_*. In both PPI and DDI tasks, most methods of individual kernel combination assign the same weight to each individual kernel [12], [20], [21], and sometimes their combined kernels fail to achieve the best performance [20], [21]. The reason is that that each individual kernel has a different performance, and only when the kernels with better performances are assigned higher weights can the combination of individual kernels produce the best result [22]. However, the manual selection of appropriate weight for each kernel is a time-consuming and imperfect art.

In our method, Stacked generalization [17] is introduced to automatically learn the weights assigned to the individual kernels from the training data. Stacked generalization is an approach for combining multiple classifiers that have been learned for a classification task. The performance of Stacked generalization is very competitive compared with arcing and bagging (which are presented by Breiman [23], [24]) [25].

Stacked generalization is a layered architecture. The classifiers at the layer-0 (*level-0*) receive the original data as their inputs, and each classifier outputs a prediction for its own sub-problem. Successive layers receive the predictions of the layer immediately preceding it as an input, and finally a single classifier at the top level outputs the final prediction. Stacked generalization attempts to minimize the generalization error by using the classifiers at higher layers to learn the type of errors made by the classifiers immediately below. Here we show how stacking with two layers (*level*) works.

**Figure 2:**
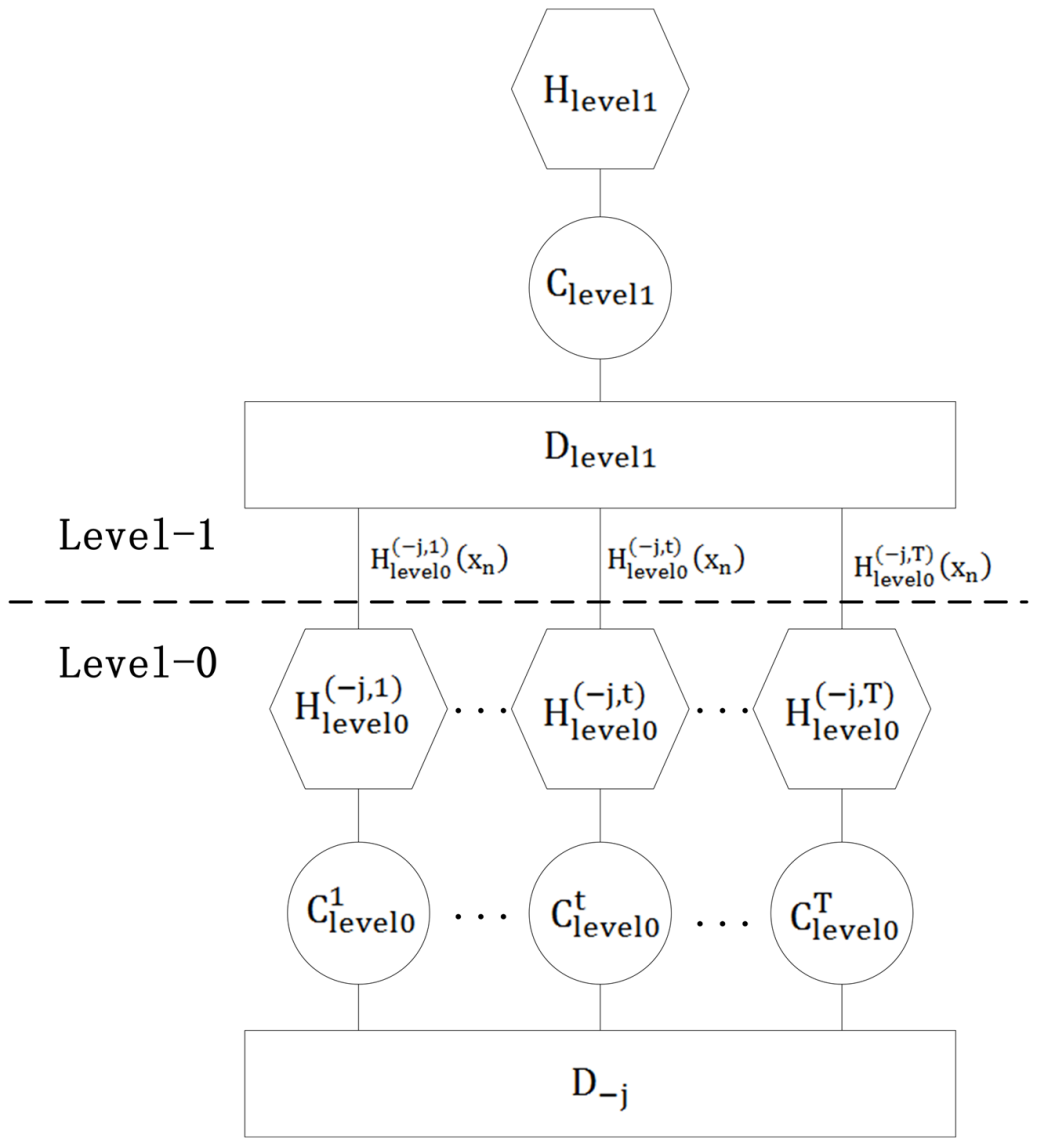
The training process of Stacked generalization. The J-fold cross-validation process in level-0; and the level-1 dataset 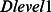 at the end of this process is used to produce the level-1 hypothesis *H*.

As shown in Figure 2, given a dataset 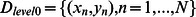, where 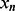 is a vector representing the attribute values of the *n*th instance and *y_n_* is the class value. Then, randomly divide 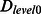 into *J* almost equal size parts 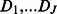 and define 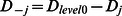, where *j = *1, …, *J*.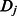 and 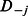 are used as the test and training sets for the *j*th fold of a *J*-fold cross-validation, respectively. There are *T* base classifiers, which we call the *level-0 generalizers*. The *t*th base classifier is trained using instances of the training data set 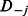 to output the hypothesis 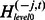, for *t* = 1, …, *T*. These are called the *level-0 hypotheses*. For each instance 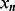 in 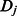, the test set of the *j*th cross-validation fold, let 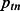 denote the prediction of the hypothesis 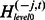 on 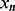. By processing the whole *J*-fold cross-validation, the level-1 training set 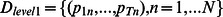 is assembled from the outputs of the *T* hypotheses. Then a classifier that we call the *level-1 generalizer* is used to derive a hypothesis 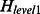 from the level-1 training data 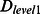. Figure 2 illustrates the training process. To complete the training process, the final level-0 hypotheses 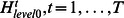, are derived using all the data in 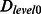.

**Figure 3:**
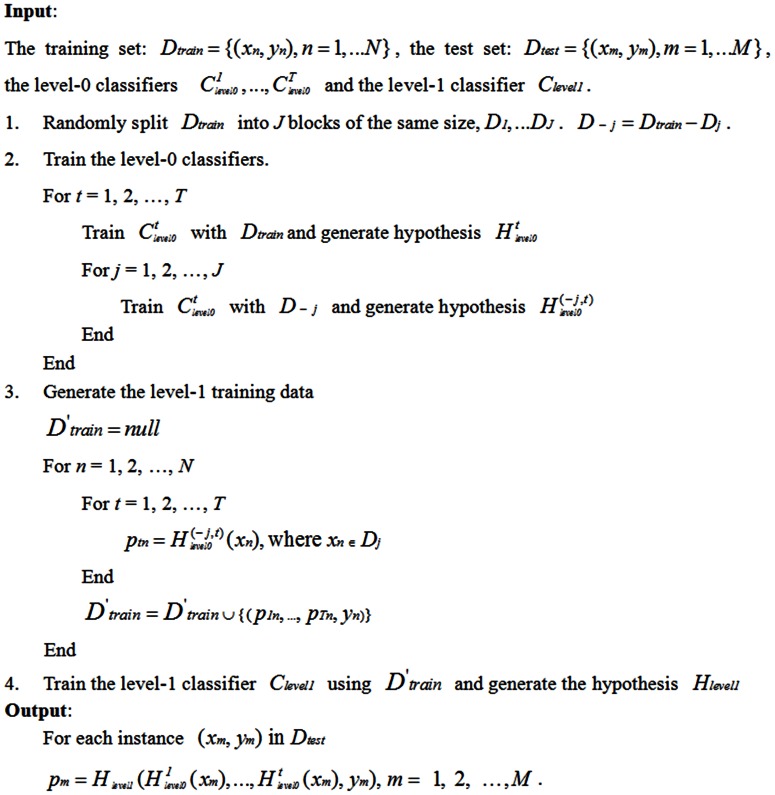
The algorithm of the stacking process.

Now let us consider the classification process, which uses the hypotheses 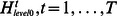, in conjunction with 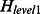. When presented with a new instance, it is first classified using 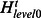. Thus an input vector 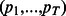 is generated and then classified using 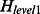, which outputs a prediction for the instance. The algorithm of the stacking process is shown in Figure 3.

In our method, the feature-based, graph and tree kernels are used as the level-0 generalizers. As for the level-1 generalizer, we compared the effects of three different learning algorithms: Multiple Linear Regression (MLR) [26], Support Vector Machines (SVM) [27] and Ranking-SVM [28]. MLR is an adaptation of a least-squares linear regression algorithm that Breiman used in regression settings [29]. Any classification problem with real-valued attributes can be transformed into a multi-response regression problem. If the original classification problem has *I* classes, it is converted into *I* separate regression problems, where the problem for class *l* has instances with responses equal to one when they have class *l* and zero otherwise. MLR was demonstrated in [25] to be more suitable for the level-1 generalizer than C4.5 (a decision tree learning algorithm), NB (a reimplementation of a Naive Bayesian classifier) and IB1 (a variant of a lazy learning algorithm that employs the p-nearest-neighbor method using a modified value-difference metric for nominal and binary attributes).

The foundations of SVM have been developed by Vapnik [27] and are gaining popularity due to their many attractive features and promising empirical performance. SVMs are binary classifiers for a set of training data 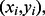 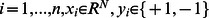 where *x_j_* is a feature vector of the *j*th training sample, and *y_j_* is the class label associated with the *j*th training sample. The decision function is defined by
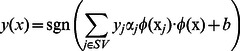
(7)where 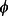 is a nonlinear mapping function from R^N^ to R^H^ (N<<H), 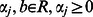, and *SV* is a set of support examples. The mapping function 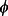 should be designed such that all training examples are linearly separable in *R^H^* space. SVMs take a maximal margin strategy in that the parameters are chosen so that the minimum distance between examples and the separating hyperplane (i.e., margin) is maximized.

Ranking SVM uses SVM for the task of pairwise classification [28]. In essence, Ranking SVM is the classification of the differences of positive and negative document pairs’ feature vectors. Compared with the regular classification SVM, Ranking SVM takes the ranking information into account, which is helpful to enhance the classification performance. In our Stacked Generalization-based approach, Ranking SVM uses the output scores of the level-0 generalizers (the feature-based, graph and tree kernels) as its features. The difference of the positive and negative instances’ scores outputted by the level-0 generalizers is used as the ranking information for Ranking SVM. In addition, when there are *M* positive examples and *N* negative examples, there are only *M*+*N* training samples for SVM, while there are M*N samples for the Ranking SVM because its samples are based on example pairs. More samples may achieve better performance.

## Results and Discussion

### Experimental Settings

Our approach was evaluated on the DDI Extraction 2011 challenge task dataset [9] and, therefore, the results can be compared with those of the participating systems. It consists of 579 documents describing DDIs. These documents are randomly selected from the DrugBank database and analyzed by the UMLS MetaMap Transfer (MMTx) tool. A total of 2402 positive instances and 21425 negative instances are identified in the training dataset (435 documents). For the test dataset (144 documents), there are 755 positive instances and 6271 negative instances.

In our implementation, we used the SVM^light^ package (http://svmlight.joachims.org/) developed by Joachims for our feature-based kernel. The linear kernel is used with a parameter c of 0.006 obtained from the 10-fold cross-validation on the training dataset, and the other parameters are default. As for the graph kernel, the all-paths graph kernel proposed by Airola et al. (http://mars.cs.utu.fi/PPICorpora/GraphKernel.html) is used. For the tree kernel, we chose Tree Kernel Toolkit developed by Moschitti with the default parameters (http://dit.unitn.it/~moschitt/Tree-Kernel.htm).

The existing DDI extraction system evaluations use the balanced F-score measure for quantifying the performance of the systems [9]. This metric is defined as F-score = (2PR)/(P+R) where P denotes the precision and R denotes the recall. Therefore, the F-score is also used in our work to measure the performance so that it can be compared with those of other existing DDI extraction systems. In addition, we provide our performance in terms of MCC (Matthews Correlation Coefficient) and AUC (Area Under roc Curve).

### Results

In this section, we discuss the effectiveness of different features used in the feature-based kernel, the effectiveness of different kernels, and the comparison of our results with those of earlier works.

#### Effectiveness of different features in the feature-based kernel

The classification performances of different features in the feature-based kernel is shown in Table 2. Among other lexical features, the Abon feature achieves the best performance (an F-score of 63.43%). When it is combined with the San and Cpn features, the F-scores are improved by approximately 1 and 1.5 percentage units, respectively, and an F-score of 64.94% is achieved. As introduced in Section *Methods*, our feature-based kernel uses five other types of features besides the lexical features. Experimental result shows that, when these five features are introduced, the overall performance is improved by 1.7 percentage units in F-score (from 64.94% to 66.63%). Among others, the keyword, semantic type, and Drugbank features employ domain knowledge and contribute to a performance improvement of 1.2 percentage units in F-score (from 65.44% to 66.63%).

**Table 2:** Performances of different features in the feature-based kernel.

Feature		Precision	Recall	F-score	MCC	AUC
Lexical features	Abon	57.40	70.86	63.43	58.92	90.7
	San	50.05	66.36	57.06	51.73	89.8
	Cpn	54.61	47.81	50.99	45.65	86.0
	Abon+San	57.20	73.64	64.39	60.11	91.2
	Abon+San+Cpn	58.49	72.98	64.94	60.67	91.8
Lexical features+Negative word		60.80	70.46	65.28	60.96	93.0
Lexical features+Negative word +NameIsDrug		59.40	72.58	65.44	61.06	92.1
Lexical features+Negative word +NameIsDrug+Keyword		59.09	73.64	65.57	61.38	92.8
Lexical features+Negative word+NameIsDrug+Keyword+Semantic type		60.88	71.52	65.77	61.63	92.7
Lexical features+Negative word+NameIsDrug+Keyword+Semantic type+Indication		59.50	73.51	65.91	61.76	92.0
Lexical features+Negative word+NameIsDrug +Keyword+Semantictype+ Indication+Pharmacology		59.87	74.70	66.47	62.42	92.2
Lexical features+Negative word+ NameIsDrug+Keyword+Semantictype +Indication+Pharmacology+Description		60.30	74.44	66.63	62.58	92.2

#### Effectiveness of different kernels

The performance of different kernels is shown in Table 3. The feature-based kernel has superior performance (66.63% in F-score) compared with the other two kernels. The key reason is that, in the feature-based kernel, in addition to the commonly used word features, the keyword, semantic type, and DrugBank features are introduced to improve the performance. The introduction of these features is a way of employing domain knowledge and is shown to improve the performance effectively. The performance (62.21% in F-score) of the graph kernel ranks second because it considers the parser’s output and word features together. Finally, the performance of the tree kernel ranks the lowest (53.06% in F-score).

**Table 3:** Performances of each individual kernel and their combinations.

Method	Precision	Recall	F-score	MCC	AUC
Feature-based kernel	60.30	74.44	66.63	62.58	92.2
Tree kernel	48.06	59.21	53.06	49.94	84.6
Graph kernel	62.84	61.59	62.21	57.71	90.3
Feature-based kernel (0.5) +Graph (0.5)	63.86	73.25	68.23	64.30	92.7
Feature-based kernel (0.33) +Graph (0.33) +Tree (0.33)	65.05	71.26	68.01	64.05	92.7
Feature-based kernel (0.45) +Graph (0.4) +Tree (0.15)	66.79	72.19	**69.38**	**65.60**	**92.9**
SVM as the level-1 classifier	62.21	70.86	68.46	62.06	92.8
MLR as the level-1 classifier	66.17	71.25	68.62	64.74	92.7
5–5 Ranking SVM as the level-1 classifier	67.18	70.99	69.03	65.21	92.7
6–4 Ranking SVM as the level-1 classifier	67.43	70.46	68.91	65.10	92.8
7–3 Ranking SVM as the level-1 classifier	66.22	72.45	69.20	65.38	92.9
8–2 Ranking SVM as the level-1 classifier	70.46	67.55	69.06	65.33	92.9
9–1 Ranking SVM as the level-1 classifier	70.39	67.68	69.01	65.37	92.9
Ranking SVM as the level-1 classifier	66.18	72.58	**69.24**	**65.43**	**92.9**

The weights of each individual kernel in combined kernels are in the parentheses after the kernel name.

In addition, the experimental results show that when the feature-based kernel is combined with the graph kernel, the performance is improved by 1.6 percentage units in F-score (from 66.63% to 68.23%). When further combined with the tree kernel, if the kernels are assigned the same weight, the overall performance will decrease from 68.23% to 68.01%; if the kernels are assigned the optimal weights (the weights are tuned on the test set and the performance achieved with them can be regarded as the best performance that could be achieved on the test set), the overall performance will improve from 68.23% to 69.38%.

The experimental results demonstrate that, on the one hand, as discussed in Section *Methods*, because the different kernels calculate the similarity between two sentences from different aspects, the combination of kernels covers more knowledge and is effective for DDI extraction; on the other hand, if combined with the same weight, the performance of the combined kernel will deteriorate owing to the introduction of an individual kernel with poor performance.

The performances of MLR, SVM and Ranking-SVM as the level-1 generalizer of Stacked generalization are also shown in Table 3. Both SVM and Ranking-SVM use the linear kernel with the parameters c of 0.1 and 10, respectively. The performances of MLR and SVM are almost the same (68.62% to 68.46% in F-score), while that of Ranking-SVM is better (69.24%) and very close to the optimal performance (69.38%) achieved with the optimal weights tuned on the test set. The performance of the stacked generalization is also the best in terms of MCC and AUC. The reason may be that, as discussed in Section *Methods*, Ranking SVM takes the ranking information into account, which is helpful to enhance the classification performance. In addition, for given training samples, Ranking SVM can have more samples because its samples are based on example pairs, and more samples may achieve better performance.

In [30], we have proved the effectiveness of Ranking SVM in combining the outputs of individual kernels in the PPI extraction task. However, the experimental method in [30] is different from the one presented in this paper. In [30], to train a Ranking SVM model, the whole corpus is partitioned into *N* datasets of equal size. The feature-based, tree and graph kernels are trained on *M* (*M*<*N*) datasets (called the training set), respectively. Then, their test results on other *L* (*L*<*N* and *L*+*M* = *N*) datasets (called the validation set) are used as the training data for the Ranking SVM model. In the Stacked generalization presented in this paper, the level-1 training set for Ranking SVM is assembled by processing the whole *N*-fold cross-validation, and its size is *N*. With more samples, it is reasonable that Ranking SVM in Stacked generalization achieves a better performance.

To further verify the idea, the experiments similar to those in [30] are conducted on the DDI Extraction 2011 corpus. The training set of DDI Extraction 2011 is divided into the training set and validation set of different sizes (5–5, 6–4, 7–3, 8–2, 9–1, respectively). Take 5–5 for an example. The training set of DDI Extraction 2011 is divided into 10 datasets of equal size. Five datasets are used as the training set, and the other five are used as the validation set. The feature-based, tree and graph kernels are trained on the training set, respectively. Then their test results on the validation set are used as the training data for the level-1 generalizer Ranking SVM model. The trained Ranking SVM model is then tested on the test set of the DDI Extraction 2011. The experimental results are also shown in Table 3. The performances of 5–5, 6–4, 7–3, 8–2, 9–1 Ranking SVM models range from 68.91% to 69.20% in F-score, which are inferior to that of Ranking SVM in the Stacked generalization presented in this paper (69.24%). What is more, in the method of [30], the sizes of the training set and validation set need to be tuned to achieve the optimal performance while it is not needed in the method of Stacked generalization.

#### Performance comparison with other methods in the DDI Extraction 2011 challenge task

As mentioned in the Section *Introduction*, several kernels have been proposed for information extraction from the biomedical literature, including the tree kernels, shortest path kernels, and graph kernels. Each kernel utilizes a portion of the structures to calculate the useful similarity, whereby any one kernel cannot retrieve the other important information that may be retrieved by other kernels. The combination of multiple kernels can gather more information and cover some of losses. Therefore, in recent years, studies have proposed the use of multiple kernels to retrieve the widest range of important information in a given sentence [31], [32].

Similarly, in the DDI Extraction 2011 challenge task, the best performing methods are also multiple kernel-based ones [10], [12] (the results are summarized in Table 4). Thomas et al. obtained an F-score of 65.74% using the combination of APG, SL and moara [10]. Their APG is the same as ours. However, its performance (63.53% F-score) on the DDI Extraction 2011 test set is better than that of our APG (62.21% F-score). The reason may be that the preprocessing methods are different: Thomas et al. used the Charniak-Lease parser with a self-trained re-ranking model augmented for biomedical texts to parse each sentence, whereas we used the Stanford parser. The SL kernel is defined as the sum of two kernels, the global context (GC) kernel (which is based on the words occurring in the sentence *fore-between*, *between* and *between-after* relative to the pair focused entities) and the local context (LC) kernel (which uses the surface (capitalization, punctuation, numerals) and shallow linguistic (POS-tag, lemma) features generated from tokens to the left and right of the entities of the entity pair) [31]. Actually, the SL kernel is similar to the feature-based kernel used in our method. However, the results of our feature-based kernel (66.63% in F-score) outperforms that of SL (60.05% in F-score). The reason is that our feature-based kernel introduces some domain knowledge based features, such as the keyword, semantic type, and DrugBank features, which prove to be effective in improving the DDI extraction performance. What’s more, the shallow syntactic features, such as POS, added to a lexical feature set are reported not to increase the performance of the classifier in [32], [33]. Moara uses case-based reasoning (CBR) for classifying the drug pairs. CBR is a machine learning approach that represents data with a set of features [34]. Our feature-based kernel (66.63% in F-score) outperforms that of Moara (44.4% in F-score) because it introduces richer features. Chowdhury et al. obtained an F-score of 63.98% by combining the feature-based method and the kernel-based method consisting of the MEDT, PST, and SL kernels [12]. The feature-based method (FBM) contains two types of features: lexical features (Word features, Morphosyntactic feature, etc.) and advanced features (Trigger word and Negation). These two types of features are also used in our feature-based kernel, and, in addition, we added other features, including domain knowledge features, which is the reason that the performance of FBM is inferior to ours. In their kernel-based method, MEDT is a version of the expanded DT kernel. DT is a dependency tree that is a representation that denotes the grammatical relations between words in a sentence [35]. The dependency path tree kernel used in our paper also uses the information derived from DT and, in some cases, it is extended to include more information as described in Section *Methods*. Their PST kernel is similar to the SPT kernel in our method, which is based on the smallest common subtree of a phrase structure parse tree including the two entities involved in a relation. The difference is that, like for the dependency path tree kernel, we also extend SPT in some cases.

**Table 4:** Performance comparison with other methods on the DDI Extraction 2011 challenge task dataset.

Methods	Precision	Recall	F-score	MCC	AUC
Thomas et al. [10]	60.54	71.92	65.74	61.50	–
Chowdhury et al. [12]	58.59	70.46	63.98	58.25	–
Our combined kernel-1	65.05	71.26	68.01	64.05	92.7
Our combined kernel-2	66.18	72.58	**69.24**	**65.43**	**92.9**

In our method, the feature-based kernel alone yields a comparable performance (66.63% in F-score) with the above two methods. With the introduction of the graph and tree kernels our method achieves even better performance. In our experiments, we tried two different multiple kernel combination methods. The first one is denoted as “Our combined kernel-1” in Table 4, which assigns the same weights to all three kernels. Its performance (68.01% in F-score) is inferior to that of “Our combined kernel-2” (69.24% in F-score), in which Stacked generalization is introduced to automatically learn the weights assigned to the individual kernels from the training data, and its performance is very close to the optimal performance (69.38%) achieved with the weights tuned on the test set.

In addition, in the DDI Extraction 2011 challenge task, Björne et al. [15], Minard et al. [16] and Garcia-Blasco et al. [36] only used the feature-based method. Björne et al. applied their open source Turku Event Extraction System, which was the best performing system in the popular BioNLP 2009 Shared Task. They adapted the Turku System to the DDI task by extending it with a new example builder module, which converts the DDI corpus into machine learning classification examples, taking into account information specific for drug-drug interactions. The features they used are tokens, dependency n-grams built from the shortest path of dependencies, path terminal token attributes and sentence word count. What’s more, they marked as a feature for each candidate pair whether it is present in DrugBank and whether it is there as a known interacting pair; they also used the information from the MMTx format dataset. In our method, we also used the information from Drugbank and the MMTx format dataset, but the information we used is much richer than theirs, as described in Section *Methods*. The experimental results show that our feature-based kernel outperforms theirs (62.99% in F-score). The reason may be that they used so many features that some noise was introduced. The shallow syntactic features such as POS added to a lexical feature set are reported not to increase the performance of the classifier, while the deep plus shallow syntactic- and lexical-feature based classifier showed a poor performance when the set of lexical features was limited [37]. Both Minard et al. and Garcia-Blasco et al. used the lexical features. Minard et al. used all the accessible information of the corpus as features, and they used the technology of information gain to select 1010 important features. Their method achieves a performance of 59.65% in F-score. Garcia-Blasco et al. used both the lexical features and the domain knowledge, such as keywords derived from the training corpus, drug semantic types, classes and drug IDs derived from the MMTx format dataset. Their method achieves a performance of 58.29% in F-score. Our feature-based kernel outperforms the above three methods because it integrates both the lexical features and domain knowledge based features, such as the keyword, semantic type, and DrugBank features.

### Error Analysis

Confined to the complexity of natural language, extracting DDIs from the biomedical literature remains a challenging task, and it is difficult to achieve a satisfactory performance. We manually analyzed 200 positive instances from the test dataset that were classified as negative instances by our classification model (i.e. false negatives. Some examples are given in Table 5). A detailed analysis of all types of false negative errors is shown in Table 6. Firstly, there is the problem of the corpus annotation consistency. For example, the instances P1, P2, P3 and P4 in Table 5 should be classified as the same class since the interaction patterns in them are exactly the same. However, according to the corpus’s annotation, P1 and P3 are labeled as positive instances while P2 and P4 are labeled as negative instances. The corpus annotation error accounts for 35.5% of the 200 false negative errors.

**Table 5:** Examples of DDI instances. The focused entities of each pair are typeset in bold.

	Instances	Our result	Corpus’s annotation
**P1**	**Amiodarone** may suppress certain CYP450 enzymes, including **CYP1A2**, CYP2C9, CYP2D6, and CYP3A4.	**negative**	**positive**
**P2**	**Amiodarone** may suppress certain cyp450 enzymes, including CYP1A2, **CYP2C9**, CYP2D6, and CYP3A4.	**negative**	**negative**
**P3**	**Amiodarone** may suppress certain cyp450 enzymes, including CYP1A2, CYP2C9, **CYP2D6**, and CYP3A4.	**negative**	**positive**
**P4**	**Amiodarone** may suppress certain cyp450 enzymes, including CYP1A2, CYP2C9, CYP2D6, and **CYP3A4**.	**negative**	**negative**
**P5**	Although not studied with alosetron, inhibition of **N-acetyltransferase** may have clinically relevantconsequences for **drugs** such as isoniazid, procainamide, and hydralazine.	**negative**	**positive**
**P6**	Intestinal **adsorbents** (e. g., charcoal) and digestive enzyme preparations containing carbohydrate-splittingenzymes (e. g., amylase, pancreatin) may reduce the effect of **Acarbose** and should not be taken concomitantly.	**negative**	**positive**
**P7**	When administered concurrently, the following drugs may interact with **beta-adrenergic receptor blocking agents**: **Anesthetics**, general: exaggeration of the hypotension induced by general anesthetics.	**negative**	**positive**
**P8**	As with some other nondepolarizing **neuromuscular blocking agents**, the time of onset of neuromuscularblock induced by NUROMAX is lengthened and the duration of block is shortened in patients receivingphenytoin or **carbamazepine**.	**negative**	**positive**
**P9**	**Quinolones**, including cinoxacin, may enhance the effects of **oral anticoagulants**, such as warfarin or its derivatives.	**positive**	**negative**
**P10**	Therefore, caution should be used when administering **CYP3A4** inhibitors with **IRESSA**.	**positive**	**negative**
**P11**	Although no drug-drug interaction studies have been conducted in vivo, it is expected that no significantinteraction would occur when **nitazoxanide** is co-administered with **drugs** that either are metabolized byor inhibit cytochrome P450 enzymes.	**positive**	**negative**
**P12**	It is recommended that if **CASODEX** is started in patients already receiving **coumarin anticoagulants**, prothrombin times should be closely monitored and adjustment of the anticoagulant dose may be necessary.	**positive**	**negative**

**Table 6:** Analysis of the false negatives.

Error cause		Error number	Error proportion (%)	Example
Annotation consistency		71	35.5	**P1**, **P3**
“Drugs” annotation error		15	7.5	**P5**
Negative word error		12	6	**P6**
DDI extraction error	Failure to extract the DDI	37	18.5	**P7**
	Unobvious DDI	65	32.5	**P8**
Totals		200	100	

Secondly, some general words (e.g., “drugs”) are sometimes labeled as drug names in both the training set and test set. To train a better model, we introduce the *NameIsDrug* feature as described in Section *Methods*. However, our classification model fails to address such a problem perfectly, and it causes 15 errors (for example, the example P5 is labeled as a positive instance while it is classified as a negative instance by our model), accounting for 7.5% of the total errors.

Thirdly, the existence of the negative words is considered to be an indicative feature used in our feature-based kernel. Therefore, when a negative word exists in an instance, it tends to be classified as a negative one. However, it may cause some false negatives as shown in the example P6. It can be observed that a negative word “not” exists in P6, and our classification model classifies it as a negative instance while in fact it is labeled as a positive one. The Negative word error accounts for 6% of the total errors.

Lastly, confined to the complexity of the DDI expression as well as the quantity and quality of the training set, many false negatives are generated, which accounts for most of the total false negative errors (51%). These DDI extraction errors can be further divided into two classes: 1) Failure to extract the DDI. Confined to the complexity of the DDI expression, our classification model fails to classify some positive instances. The example P7 is such as an example. 2) Unobvious DDI. In some DDI instances (e.g. the example P8), the DDI relationships are rather unobvious and it is even difficult for a human being to determine whether they are positive or negative instances. We provide more examples of the false negatives due to the DDI extraction error in the supplementary table S1.

In addition, 200 negative instances classified by our model as positive instances were also manually analyzed. A detailed analysis of all types of false positive errors is shown in Table 7. Firstly, in some instances, the annotated drug names are the general names of a kind of drug names. For example, in the example P9, *Quinolones* are a family of synthetic broad-spectrum antibiotics, which includes *cinoxacin*. Our model can not differentiate it from a specific drug name (e.g. *cinoxacin*) and classifies the DDI as a positive instance while it is labeled as a false instance in the corpus’s annotation.

**Table 7:** Analysis of the false positives.

Error cause	Error number	Error proportion (%)	Example
General drug name error	48	24	**P9**
Non-drug name annotation error	26	13	**P10**
“Drugs” annotation error	12	6	**P11**
DDI extraction error	114	57	**P12**
Totals	200	100	

Secondly, in some other instances, the annotated drug names are not true drug names. For example, in the example P10, one of the annotated drugs, CYP3A4, is a gene. Like in the example P9, our model can not differentiate it from a true drug name and classifies the DDI as a positive instance.

Thirdly, as discussed in the previous part of this section, the word “drugs” is sometimes labeled as drug names in both the training set and test set. Our model can not address such a problem perfectly and classifies some negative instances as positive instances as shown in the example P11.

Lastly, confined to the complexity of the DDI expression, our model fails to classify some true negative instances correctly, which accounts for most of the total false positive errors (57%). Since we can not further expand the DDI extraction error reason for these false positive instances, we provide more examples of the false positives due to the DDI extraction error in in the supplementary table s2.

### Conclusions

Because side effects of drugs can be very dangerous, DDI detection is the subject of an important field of research that is crucial for both patient safety and the control of health care costs. Although health care professionals can perform DDI extraction using different databases, those being used currently are rarely complete because their update periods can be as long as three years [38].

In this paper, we present a Stacked generalization-based approach for automatic DDI extraction. The approach introduces Stacked generalization to automatically learn the weights from the training data and assigns them to three individual kernels (feature-based, tree and graph kernels), achieving a much better performance than each individual kernel. This indicates that the features in the individual kernels are complementary and can be successfully combined with Stacked generalization due to: 1) the flat entity information captured by the feature-based kernel; and 2) the structured syntactic connection information between the two entities captured by the tree and graph kernels. Experimental results show that our method can achieve the better performance with respect to comparable evaluations, with an F-score of 69.24% on the DDI Extraction 2011 challenge task corpus.

According to our experience with information extraction from the biomedical literature, among others, the feature-based, tree and graph kernels are three typical kernels. Each kernel calculates the similarity between two sentences from different aspects, and thus combining the similarities can reduce the risk of missing important features. However, the selection of an appropriate weight for each kernel manually is a time-consuming and imperfect art. To solve the problem, Stacked generalization can be introduced to automatically learn the weights assigned to the individual kernels from the training data, whose performance is very competitive compared with arcing and bagging [25]. As the level-1 generalizer of Stacked generalization, the performance of Ranking-SVM is better than those of MLR and SVM, and it is very close to the optimal performance achieved with the weights tuned on the test set because it takes the ranking information into account, which is helpful to enhance the classification performance.

In addition, our experimental results show that, as a way of employing domain knowledge, the introduction of the keyword, semantic type, and DrugBank features is effective in improving the DDI extraction performance. Therefore, the introduction of more appropriate domain knowledge into DDI extraction is one important problem to be studied further.

## Supporting Information

Table S1 **Examples of the false negatives due to the DDI extraction error.** The focused entities of each pair are typeset in bold.
(DOC)

Table S2 **Examples of the false positives due to the DDI extraction error.** The focused entities of each pair are typeset in bold.
(DOC)
