# Supplementary material for: Extracting Drug-Drug Interaction from the Biomedical Literature Using a Stacked Generalization-Based Approach
Source: PLoS One. 2013 Jun 13;8(6):e65814. doi: 10.1371/journal.pone.0065814 (PMC3681788; doi:10.1371/journal.pone.0065814)
Supplement: Table S1 — Examples of the false negatives due to the DDI extraction error. The focused entities of each pair are typeset in bold. (DOC) [file pone.0065814.s001.doc]

Table S1. Examples of the false negatives due to the DDI extraction error. The focused entities of each pair are typeset in bold.

|  | **Instances** |
| --- | --- |
| **P1** | In addition to the interactions noted above, chronic (2 weeks) oral **Cordarone** administration impairs metabolism of phenytoin, dextromethorphan, and **methotrexate**. |
| **P2** | Other drugs which may enhance the neuromuscular blocking action of nondepolarizing agents such as **NUROMAX** include certain antibiotics (e. g., aminoglycosides, tetracyclines, bacitracin, polymyxins, lincomycin, clindamycin, colistin, and **sodium** colistimethate), magnesium salts, lithium, local anesthetics, procainamide, and quinidine. |
| **P3** | Pharmacokinetic interactions between **nisoldipine** and beta-blockers (atenolol, **propranolol**) were variable and not significant. |
| **P4** | **Erythromycin** (500 mg t.i.d) produced a 4-fold increase in **vardenafil** AUC and a 3-fold increase in Cmax when co-administered with Vardenafil 5 mg in healthy volunteers. |
| **P5** | When used in therapeutic doses, **azithromycin** had a modest effect on the pharmacokinetics of atorvastatin, carbamazepine, **cetirizine**, didanosine, efavirenz, fluconazole, indinavir, midazolam, rifabutin, sildenafil, theophylline (intravenous and oral), triazolam, trimethoprim/sulfamethoxazole or zidovudine. |
| **P6** | Cimetidine, Ranitidine: In normal volunteers (n=9), pretreatment with **cimetidine** or ranitidine did not affect flurbiprofen pharmacokinetics except that a small (13 %) but statistically significant increase in the area under the serum concentration curve of **flurbiprofen** resulted with cimetidine. |
| **P7** | Poor metabolizers of debrisoquin: Interactions of carvedilol with strong inhibitors of CYP2D6 (such as quinidine, fluoxetine, paroxetine, and **propafenone**) have not been studied, but these drugs would be expected to increase blood levels of the R(+) enantiomer of **carvedilol**. |
| **P8** | Avoid the use of preparations such as decongestants and local anesthetics which contain any **sympathomimetic amine** (e.g., epinephrine, norepinephrine), since it has been reported that **tricyclic antidepressants** can potentiate the effects of catecholamines. |
| **P9** | As with some other nondepolarizing **neuromuscular blocking agents**, the time of onset of neuromuscular block induced by NUROMAX is lengthened and the duration of block is shortened in patients receiving phenytoin or **carbamazepine**. |
| **P10** | The effect may be mediated by the known inhibition of **cimetidine** on hepatic cytochrome P-450, the enzyme system probably responsible for the first-pass metabolism of **nifedipine**. |
