# Supplementary material for: Extracting Drug-Drug Interaction from the Biomedical Literature Using a Stacked Generalization-Based Approach
Source: PLoS One. 2013 Jun 13;8(6):e65814. doi: 10.1371/journal.pone.0065814 (PMC3681788; doi:10.1371/journal.pone.0065814)
Supplement: Table S2 — Examples of the false positives due to the DDI extraction error. The focused entities of each pair are typeset in bold. (DOC) [file pone.0065814.s002.doc]

Table S2. Examples of the false positives due to the DDI extraction error. The focused entities of each pair are typeset in bold.

|  | **Instances** |
| --- | --- |
| **P1** | Administration of **thiazide diuretics** to hypoparathyroid patients who are concurrently being treated with **dihydrotachysterol** may cause hypercalcemia. |
| **P2** | Long Acting Nitrates: **Nifedipine** may be safely co-administered with **nitrates**, but there have been no controlled studies to evaluate the antianginal effectiveness of this combination. |
| **P3** | When atropine and pralidoxime are used together, the signs of atropinization (flushing, mydriasis, tachycardia, dryness of the mouth and nose) may occur earlier than might be expected than when atropine is used alone because **pralidoxime** may potentiate the effect of **atropine**. |
| **P4** | (**Indomethacin**) **diuretics** are used concomitantly, the patient should be observed closely to determine if the desired effect of the diuretic is obtained. |
| **P5** | **INDOCIN** can reduce the antihypertensive effects of captopril and **losartan.** |
| **P6** | Although there is little published information on concomitant administration of **lidocaine** and **Bretylium Tosylate**, these drugs are often administered concurrently without any evidence of interactions resulting in adverse effects or diminished efficacy. |
| **P7** | The following precautions should be kept in mind in the treatment of anticholinesterase poisoning although they do not bear directly on the use of **atropine** and **pralidoxime**. |
| **P8** | Serum concentration of **digoxin** and **digitoxin** may increase when patients take antithyroid agents. |
| **P9** | Symptoms resolved when **clonidine** was withdrawn and recurred when the patient was rechallenged with **clonidine.** |
| **P10** | Drugs highly bound to **albumin** could increase the unbound fraction of **fosphenytoin.** |
